# Botany as an Ally of Medicine

**Published:** 1860-02

**Authors:** 


Botany as an Ally of Medicine. A Lecture delivered to the Medical Society of the University of Nashville, Dec. 2d, 1859. By George S. Blackie, M. D., A. M., T. B. S. E., etc. p. p. 24. Nashville.

After the perusal of this interesting lecture, the reader is surprised that Botany should be regarded with so much indifference by the great mass of mankind; and it is no less surprising than true, that even intelligent physicians should be so generally ignorant of the source of four-fifths of their most valued remedial agents. But it is not so much the value of botany in determining the use of plants, as articles of the materia medica, or of diet, that is referred to by the lecturer; but it is valuable as a means of mental discipline, and important in preparing the student for the reception of more practical instruction. But among the many ways in which Botany is an "Ally of Medicine," the following is the most striking:

"The perfection of the microscope, and other means of physical diagnosis, have brought to our knowledge the fact that many diseases to which our system is liable are caused by the presence of minute vegetable organisms preying upon it. The labors of Robin Goodsir, Leidy, Ehrenberg, Berkely and others have demonstrated incontestibly this fact, which any of you can confirm for yourselves. M. Robin in his treatise on vegetable parasites, enumerates and describes no fewer than eighty-six species, *Agæ* and *Fungi*, growing upon man and the lower animals. Of course they act as foreign bodies, and give rise to diseases of various kinds. Thus in the aphthous disease of new born children termed *muguet* by the French, the tongue and cavity of the mouth are found covered with a yellow flocculent matter, in which sporules and confervoid filaments (*Oidium albicans*), in an advanced state of development, may be detected in considerable numbers. Again, vegetable fungi, such as various kinds of *torulæ*, more especially one resembling a wool-sack, first described by Mr. Goodsir, and named by him the *Sarcena ventriculi*, are found in vomited matters in certain diseases, and many even themselves give rise to disease, as certain recent cases of intestinal parasites seem to demonstrate. Then again the crust of *favus* is formed of a capsule of epidermic cells lined by a mass of fine granules from which millions of cryptogamic plants, (Achorion Schoenleini) spring up and fructify producing their spores and increasing the disease, their parasitic presence being the pathognomonic character of scald-head or ring-worm, and their sporules and offshoots entering between the fibres of the hair choke it up and cause atrophy of the bulb, of which baldness is the result. Other instances might be cited, but these will suffice. Now the fact that these diseases are of vegetable origin, and caused by spores requiring all the necessaries to vegetable existence for their development and increase, is of enormous importance in the treatment. It leads us to inquire what will contribute to the life or death of a similar vegetable spore, and to employ in our outward application the substances which will cause its death. Thus the philosophic treatment, founded on botanical knowledge, of scald-head, will be to apply emollient washes to the scalp, gradually to remove the incrustations, to remove with as little violence as possible the dead and wounded hairs in which the spores are lodged, and to apply poultices or ointments which will cover the scalp, and filling up the interstices, prevent air and moisture from causing an increase of the plant. The philosophic treatment of ring-worm will be to apply castor-oil or some such substance to part effected, and similar treatment will be found for all similar affections."

